# Responses of Upland Cotton (*Gossypium hirsutum* L.) Lines to Irrigated and Rainfed Conditions of Texas High Plains

**DOI:** 10.3390/plants9111598

**Published:** 2020-11-18

**Authors:** Addissu. G. Ayele, Jane K. Dever, Carol M. Kelly, Monica Sheehan, Valerie Morgan, Paxton Payton

**Affiliations:** 1 Department of Agricultural Sciences, College of Agriculture, Family Sciences, and Technology, Fort Valley State University, Fort Valley, GA 31030, USA; Addissu.ayele@fvsu.edu; 2 Texas A&M AgriLife Research and Extension Center, Lubbock, TX 79403, USA; cmkelly@ag.tamu.edu (C.M.K.); m-bellow@tamu.edu (M.S.); vmorgan@ag.tamu.edu (V.M.); 3 USDA-ARS Cropping Systems Research Laboratory, Lubbock, TX 79415, USA; paxton.payton@ars.usda.gov

**Keywords:** rainfed, irrigated, *Gossypium hirsutum*

## Abstract

Understanding drought stress responses and the identification of phenotypic traits associated with drought are key factors in breeding for sustainable cotton production in limited irrigation water of semi-arid environments. The objective of this study was to evaluate the responses of upland cotton lines to rainfed and irrigated conditions. We compared selected agronomic traits over time, final yield and fiber quality of cotton lines grown in irrigated and rainfed trials. Under rainfed conditions, the average number of squares per plant sharply declined during weeks 10 to 14 while the average number of bolls per plant significantly reduced during weeks 13 to 15 after planting. Therefore, weeks 10 to 14 and weeks 13 to 15 are critical plant growth stages to differentiate among upland cotton lines for square and boll set, respectively, under drought stress. Variation in square and boll set during this stage may translate into variable lint percent, lint yield and fiber properties under water-limited conditions. Lint yield and fiber quality were markedly affected under rainfed conditions in all cotton lines tested. Despite significantly reduced lint yield in rainfed trials, some cotton lines including 11-21-703S, 06-46-153P, CS 50, L23, FM 989 and DP 491 performed relatively well under stress compared to other cotton lines. The results also reveal that cotton lines show variable responses for fiber properties under irrigated and rainfed trials. Breeding line 12-8-103S produced long, uniform and strong fibers under both irrigated and rainfed conditions. The significant variation observed among cotton genotypes for agronomic characteristics, yield and fiber quality under rainfed conditions indicate potential to breed cotton for improved drought tolerance.

## 1. Introduction

Climatic variability and elevated levels of greenhouse gases could cause the induction of flooding, heat waves and drought stress [1]. Among these environmental factors, water scarcity, which leads to drought stress, is the major limitation for crop production. Water availability is a key element for sustainable cotton production and its limitation adversely affects the biochemical and physiological process of a plant, leading to a reduction of yield and fiber quality [1]. The productivity of agricultural land is seriously affected by the change in patterns of temperature, the amount and distributions of rainfall and climate change. These changes are likely to remain critical barriers to keep up with food and fiber production in the future [2,3]. As water is a limited resource, and drought frequency and intensity show increasing trends [4], appropriate use of irrigation water is expected to balance food demands with the anticipated increase of the world population growth. To succeed with the subsequent estimates of water shortages, measures aimed at reorganizing and optimizing the efficiency of water consumption in the agricultural sector are critical.

Cotton (*Gossypium* spp.) is one of the world’s most important crops, accounting for around 35% of all-natural and man-made fibers produced in the world [5]. Production of cotton in many regions of the U.S. cotton belt is limited by insufficient irrigation water and erratic rainfall patterns. In the Texas High Plains, the Ogallala aquifer historically provides irrigation water for cotton production. However, the Ogallala aquifer water table has declined by more than 50%, mainly due to the intensification of irrigated crop production [6,7,8]. Depletion of groundwater and high energy costs associated with pumping water to the surface affect cotton production, which makes the selection for drought tolerance a primary objective of cotton breeding in the high plains of Texas.

Studies indicate that when a cotton population is subjected to abiotic stresses, particularly drought and high-temperature stresses, more than 50% yield reduction occurs as compared to irrigated plants with a similar genetic background [9]. Most crops, including cotton, are sensitive to drought stress, particularly during flowering through seed developmental stages [10]. Cook and El-Zik, 1992, [11] suggested drought stress during anthesis can result in a reduction of lint yield due to shedding squares and young bolls. Drought reduces yield and fiber quality, costing producers millions of dollars each year. Wang, et al., 2016, [12] observed when soil moisture decreased from approximately 60 to 45% of field capacity, yield reduction was doubled (from approximately 30 to 60%), and fiber quality, particularly fiber length and strength, was reduced. Periodic drought also increased within-plant variability of fiber maturity and fiber length of upland cotton cultivars, being more pronounced when boll setting was in the higher fruiting branches [13].

Water availability has a direct effect on plant growth. Drought stress decreases plant turgor potential, inhibiting normal plant functions [14], and reduces both cotton yield and fiber quality [15]. Though plants with fewer bolls can compensate to some degree by producing larger bolls, the number of bolls per unit area is the most significant yield component [16]. The impact of drought stress on cotton is complex. Therefore, research is needed to better understand the responses of upland cotton to drought stress on reproductive growth, yield and fiber quality, and how they can be improved. We hypothesized that differences in response to drought stress among upland cotton genotypes exist and can lead to the identification of novel sources of germplasm that could be used for introgression of enhanced stress tolerance alleles by conventional breeding. The main objective of this study was to investigate the response of upland cotton genotypes to rainfed and irrigated conditions with respect to fiber quality, yield and reproductive growth over time, and to characterize agronomic traits useful in plant selection under drought conditions.

## 2. Results

### 2.1. The Response of Cotton Genotypes for Yield and Agronomic Traits in Irrigated and Rainfed Trials

A significant interaction effect between genotype × week was observed for the number of the squares (NSQR), number of bolls per plant (NB) and number of flowers (NF) in the irrigated trial. The NB and NF were also affected by genotype × week interaction in rainfed trial, indicating that the response of some genotypes was not the same across weeks for these agronomic traits (Table 1). Conversely, no significant effect of genotype × week interaction was observed for the number of main-stem nodes (NN) and plant height (PH) in both irrigated and rainfed trials. Genotypes showed consistent variability across weeks in rainfed conditions for NSQR (Table 2). The results revealed high variability among genotypes for NB, NSQR and PH. However, no significant differences were observed among genotypes for NF and NN in both irrigated and rainfed conditions. 

The least-square means indicate high variability among cotton lines for final yield, lint percent (LT %), boll size, plant height, and number of bolls in both irrigated and rainfed trials. Under rainfed conditions cotton lines 06-46-153P, 11-21-703S, 12-8-103S, CS 50, L 23, DP 491, and FM 989 produced a similar number of bolls per plant (Table 2). Relatively, a low average number of bolls were recorded for TX 62, TX 1151, and L23 in rainfed trials. TX 62 consistently showed poor boll setting under both irrigated and rainfed conditions. Cotton line L23 produced a good number of bolls under irrigated conditions, with poor boll set under rainfed trials. The smallest boll size was obtained from CS 50 in irrigated and rainfed trials.

Under rainfed trials, some cotton lines such as, 11-21-703S, 06-46-153P, CS 50, FM 989, and DP 491, which produced a relatively high number of bolls, tended to produce better lint yield (Table 3). The TX 1151 cotton line was the tallest plant among the cotton lines evaluated. However, TX 1151 produced the lowest NB, boll size, lint percent and lint yield both in irrigated and rainfed trials. 

**Figure 1:**
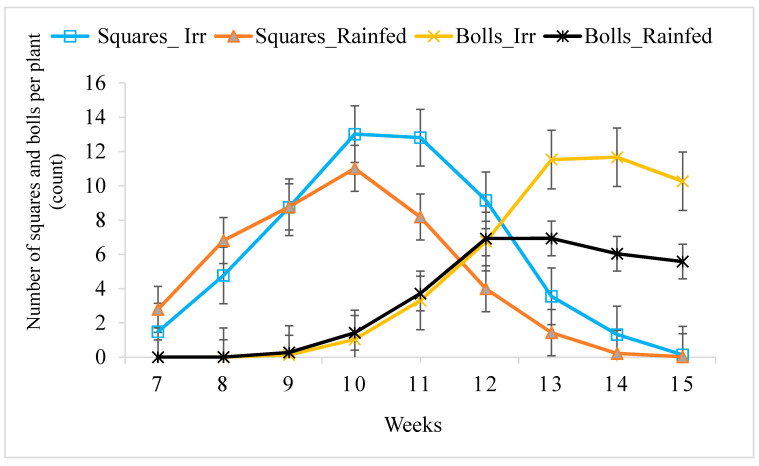
The average number of squares and bolls per plant distribution by week for upland cotton lines grown under irrigated and rainfed conditions. Data for agronomic traits were collected for nine consecutive weeks (weeks 7 to 15). Data averaged for three years, nine genotypes, and over four replications. Standard error (SE) bars were used to show the variations between irrigated and rainfed trials. Overlapping SE bars show no significant differences between irrigated and rainfed treatments.

Figure 1 depicts the effect of irrigation and rainfed treatment on the number of squares and boll retention capacity of cotton lines across weeks and growth stages. No significant differences were observed between rainfed and irrigated plots during weeks seven to 10 for the average number of squares produced per plant. In rainfed trials, the average number of squares produced per plant was significantly reduced between weeks 10 (70 DAP) to 12 (84 DAP), while in the irrigated trials, the number of squares remains constant between weeks 10 (70 DAP) to 11 (77 DAP). During week 13 (91 DAP) to 15 (105 DAP), square production sharply declined and the difference between rainfed and irrigated plots was negligible. Boll setting began around week nine after planting (Figure 1) and continuously increased until it reached a plateau during weeks 12 to 13 in rainfed, and weeks 13 to 14 after planting in the irrigated trials. The average boll setting and retention capacity of upland cotton in both irrigated and rainfed did not show differences until weeks 12 (84 DAP).

**Figure 2:**
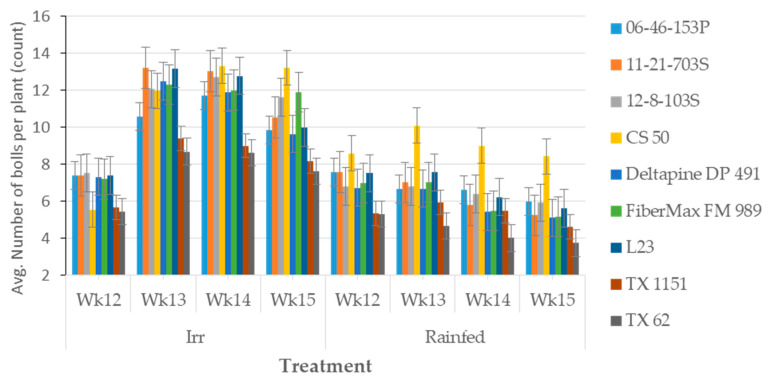
Variation in average number bolls per plant across weeks for upland cotton grown under irrigated and rainfed conditions. Wk., weeks; Irr., irrigated. Average number of bolls compared by subsampling of weeks 12 (Wk12) to weeks 15 (Wk15). Standard error (SE) bars were used to show the variations among genotypes in irrigated and rainfed trials. Overlapping SE bars show no differences between genotypes in irrigated and rainfed conditions.

Figure 2 illustrates the average number of bolls distribution per plant for different cotton lines from weeks 12 to 15 which corresponds to 84 to 105 days after planting (DAP). Cotton lines responded differently to irrigated and rainfed conditions for boll setting and retention capacity during weeks 13 (91 DAP), 14 (98 DAP) and 15 (105 DAP) plant growth stages. In rainfed trials, during weeks 13 to 15 after planting, boll production and retention capacity of CS 50 were significantly higher compared to other cotton lines. Cotton lines 06-46-153P, L23, 12-8-103S, and 11-21-703S produced a relatively higher number of bolls that were stable across weeks 13 to 15 after planting. Drought stress during weeks 13 to 15 significantly affected boll setting and retention capacity that led to variable responses among upland cotton lines. Genotypes also showed variable responses for the number of bolls per plant under rainfed conditions during weeks 13 to 15 after planting.

### 2.2. Responses of Cotton Genotypes for Fiber Quality under Rainfed and Irrigated Conditions

All high-volume instrument (HVI) measured fiber properties tested were significantly affected due to differences among cotton lines. All genotypes produced stable fiber properties relative to other genotypes in both irrigated and rainfed trials. Best performing cotton lines for fiber quality in irrigated trials were also best in rainfed trials. Cotton lines that produced poor fiber quality under rainfed produced poor fiber quality under irrigated conditions. The least-square means analysis indicates genotypes showed high variability for HVI fiber properties in both rainfed and irrigated conditions (Table 3). Compared to other cotton lines, breeding line 12-8-103S produced significantly longer and stronger fibers under both irrigated and rainfed conditions. The 12-8-103S cotton line was developed in the Texas A&M AgriLife breeding program and selected for salt tolerance. Cotton lines TX 1151, 11-21-703S and 12-8-103S produced relatively uniform fibers, while L23 and 12-8-103S produced strong fibers. TX 62, L23, 12-8-103S and DP 491 produced relatively higher elongation as compared to other cotton lines evaluated under both irrigated and rainfed trials (Table 3).

### 2.3. Correlation Analyses

Correlation results for this set of cotton lines revealed a significant and positive relationship between yield and HVI fiber properties, including micronaire (r = 0.50), fiber length (r = 0.30), length uniformity (r = 0.51), strength (r = 0.53) and elongation (r = 0.40) under rainfed conditions (Table 4). In the irrigated trial, fiber length (r = 0.29) and strength (r = 0.38) positively correlated with lint yield. The genotypes selected for this study showed a positive correlation between yield and fiber quality across growing seasons. Relatively poor yielding NCGC accession TX 62 (Table 2), selected for differential response in previous (unpublished) root development screening studies, also produced lower quality fiber (Table 3). Two breeding lines selected for salt tolerance showed relatively good yield in irrigated (12-8-103S) and rainfed (11-21-703S) trials (Table 2) and produced higher fiber quality (Table 3). Inference on correlation between yield and fiber quality was restricted to this set of lines, though results showed promise for developing lines that maintain fiber value in limited water environments. Compared to irrigated trials, a strong and positive relationship was observed between yield, agronomic traits and fiber properties of cotton produced under rainfed conditions. In the rainfed trial, a significant and positive relationship was observed between yield and final plot average agronomic characteristic such as BS (r = 0.41), NSQR (r = 0.45), NN (r = 0.57), PH (r = 0.72), and NB (r = 0.74). Plants with more NSQR, NN and NB were more productive, which indicates that these agronomic traits at certain growth stages could be used as reliable selection criteria to develop drought-tolerant cotton lines.

## 3. Discussion

Plants respond continuously to changes in various abiotic factors [17], of which the response of plants to limited water availability is considerably high. Studies show drought stress can prevent crops from reaching their genetic potential for yield, quality and agronomically valuable traits [3]. Our results also showed the potential of cotton lines to produce squares, flowers, bolls and main-stem nodes was negatively affected under rainfed conditions. Drought stress limits cotton physiological traits including photosynthesis rate and stomatal conductance leading to reduced productivity by adversely affecting valuable agronomic properties and yield of cotton [18,19]. Our results revealed high variability among selected cotton lines for agronomic characteristics, including the number of squares, bolls and flowers at different plant growth stages, and the number of main-stem nodes, leading to differences in yield and fiber quality under rainfed and irrigated conditions. The variability among agronomic characteristics, including growth and reproductive traits in upland cotton has been observed when water is a limiting factor [20,21,22,23,24]. However, limited information is available that indicates at what particular plant growth stages water stress could contribute to maximum variability among cotton genotypes for the agronomic traits most likely to affect the productivity of cotton under water-limited environments. Our results indicate that drought stress significantly affected the performances of upland cotton lines to produce squares, flowers and bolls during weeks 10 (70 DAP) through weeks 15 (105 DAP), which resulted in reduced boll size, lint turnout and lint yield.

When soil moisture is depleted, young bolls tend to shed [25]. In this study, drought stress tended to accelerate square shedding of cotton lines particularly during weeks 10 to 12 after planting. Similarly, the number of bolls set was significantly reduced during weeks 13 to 15 after planting in the rainfed trials, compared to the number of bolls set in the same period under irrigated conditions. The negative impact imposed by drought stress on the valuable agronomic traits at these critical plant growth stages may result in reduced lint yield and fiber quality.

Studies indicate that the number of bolls formed under drought is less than the number of bolls produced during the nonstress growing seasons [23,26]. We observed that drought stress not only reduced the number of bolls overall but also revealed potential variability among cotton lines at some plant growth stages. Under rainfed trials, the maximum variability of cotton boll production among cotton lines was recorded during weeks 13 to 15 after planting. For example, the CS 50 cotton line produced a relatively higher number of bolls in rainfed trials. Under drought stress conditions, cotton lines 06-46-153P, L23, 12-8-103S, and 11-21-703S produced a relatively higher number of bolls that were stable across weeks 13 to 15 after planting. Some cotton lines, such as TX 1151 and TX 62 produced a significantly low number of bolls under rainfed conditions. Among cotton lines evaluated, 11-21-703S, 06-46-153P, CS 50, L23, FM 989, and DP 491 produced higher lint yield under rainfed conditions.

Our findings, like many others, indicate that, generally, differences in yield loss observed among cotton genotypes may be attributed to the severity and duration of drought at critical plant growth stages The variability observed among genotypes for the number of squares and bolls set per plant at 10–15 weeks after planting, boll size and lint turnout tended to follow the variability observed in lint yield. Therefore, evaluating breeding nurseries based on the relative number of squares and bolls set at weeks 10 to 14 and weeks 13 to 15, respectively, may be predictive of differences in yield potential among genotypes under water-limited environments of the Texas high plains.

All HVI fiber properties showed significant variability in response to irrigated and rainfed trials. Genotypes showed consistent variability for fiber quality in both irrigated and rainfed conditions, which means that cotton lines with better performance under irrigation also performed well in rainfed trials. Similarly, genotypes with low fiber quality in the irrigated trials also produced low fiber quality under rainfed trials. 

Studies indicate that drought stress has negative effects on fiber properties, including fiber length, fiber fineness, fiber strength and fiber elongation [25,27,28]. Cotton lines show variable responses for fiber properties under irrigated and rainfed trials. For example, breeding line 12-8-103S produced long, uniform and strong fibers in both rainfed and irrigated trials and is a good candidate for further research on improving fiber quality. 

As in the results obtained by [29], we observed a significant and positive relationship between lint yield and other agronomic properties. The author of [23] also observed that yield components and agronomic traits were positively associated with yield in a drought-stressed condition. In this study, boll size, number of bolls and number of nodes showed a significant and positive association with lint yield. For cotton lines included in this study, the results did not necessarily agree with the previous studies that indicated fiber quality traits are negatively associated with fiber yield [30,31]. However, a significant and positive relationship between yield and fiber length (r = 0.61), length uniformity (r = 0.64), and strength (r = 0.59) was observed among the lines selected for this study, which indicates the possibility of simultaneous improvement of cotton for yield and fiber quality. In addition to evaluating cotton for square and boll development at critical plant growth stages to help select for yield potential under drought stress conditions, it is important to select for high fiber quality so that cotton fiber value can be retained in limited water production. The variation observed among genotypes for different fiber properties in rainfed conditions reveals the possibility of selection for genotypes that can produce adequate fiber properties for water-limited cotton production in the high plains of Texas.

## 4. Materials and Methods 

### 4.1. Plant Materials

Field trials were conducted during the 2014, 2015 and 2016 growing seasons at the Texas A&M AgriLife Research and Extension Center at Lubbock (LREC) on Olton Clay loam soil (fine, mixed, superactive, thermic Aridic Paleustolls). LREC is located at 33°41′ N, 101°49′ W, the elevation is 997 m above sea level and the average annual rainfall is 472 mm. Lubbock is characterized by a semiarid climate, resulting in dry conditions with low precipitation (Table 5), which provides a good environment to study crop drought response. From an initial screening of several genotypes, nine cotton lines were selected to evaluate phenotypic response under irrigated and rainfed conditions: three LREC breeding lines (06-46-153P, 11-21-703S, 12-8-103S), cultivars Deltapine DP 491 (PI 618609), FiberMax FM 989 (PI 639508), CS 50 and SIOKRA L23 [32], and two accessions from the National Cotton Germplasm Collection (NCGC), TEX 1151 (PI 529967) and TX 62 (PI 154096). DP 491 and FM 989 are cultivars that have been successfully grown in commercial production in Texas. The breeding line 06-46-153P was developed in the LREC cotton breeding program and registered as CA 4007 [33]. For this study, breeding lines and cultivars were selected based on differential response to multilocation performance testing over years in irrigated and rainfed trials in West Texas. NCGC accessions were selected for a variable response to greenhouse-observed root development. The selected cotton lines represented variation in maturity, plant height, yield, and fiber quality. 

### 4.2. Experimental Design and Agronomic Practices 

Within the irrigated and rainfed field trials, upland cotton genotypes were arranged in a randomized complete block design (RCBD) with four field replications. Seeds were planted in 4-row 12.19 m long plots on 1.02 m wide centers, each with 10.16 cm spacing between plants. Plants were managed under two conditions, rainfed and irrigated. Plants under irrigated conditions were managed within LREC irrigation capacity to apply water to sustain the growth and development of irrigated cotton on the Texas high plains. Irrigation water was usually applied on a monthly interval if there was no rain during the growing season. Irrigation water was delivered until the furrows were full and the duration of each irrigation time was recorded. The irrigation volumes and depth were estimated using the known flow rate of the irrigation pump. Note that rainfall in West Texas is not evenly distributed and varies from year to year. Under the rainfed conditions, plants did not receive any supplemental irrigation throughout the growing season. However, in both growth conditions, pre-irrigation was applied for all growing seasons to initiate seed germination. In 2014, total water applied, including precipitation to the irrigated trial, was 505 mm, while rainfed trials received 348 mm from precipitation. In 2015, the total volume of water applied to irrigated trials was 546 mm, while rainfed received 208 mm from precipitation. In 2016, the total volume of water applied to the irrigated trial was 636 mm while the rainfed trial received 316 mm water obtained as rainfall (Table 5). In 2014, cotton was planted on 19 May and harvested on 20 November. In 2015, cotton was planted on 27 May and harvested on 3 November for the rainfed and 10 November for the irrigated trials. In 2016, cotton was planted on 13 May and harvested on 15 November. Fertilizer was applied preplant incorporated at the rate of 80-0-0 kg N-P2O5-K2O ha^−1^ for both irrigated and rainfed experimental plots throughout the growing seasons. All other in-season agronomic inputs, such as applications of herbicide and insecticide, were applied following agronomic practices typical for cotton production for Lubbock County. Accumulated growing degree days (GDD15.6) were calculated as the average of the daily maximum and minimum air temperatures less than the base temperature of 15.6 °C [34].

### 4.3. Data Collection 

In the 2014 and 2015 growing seasons, plant height (PH), number of squares (NSQR), number of flowers (NF), number of main-stem nodes (NN) and number of bolls (NB) per plant were recorded for nine consecutive weeks to understand the response of genotypes at different growth stages under irrigated and rainfed conditions. In 2014 and 2015, the first week of data recorded in the growth stages began around 50 DAP (weeks 7) and was completed at 107 DAP (weeks 15). In both growing seasons, a set of 10 plants in each plot were tagged to measure all agronomic traits for nine consecutive weeks. Plant height was recorded from the base of the plant to the meristematic leaf at the apical bud using a measuring tape. The number of bolls per plant was an average of bolls from 10 plants. In 2016, the field data collection strategy was modified based on the 2014 and 2015 evaluations. In 2016, data for different agronomic traits were collected for three consecutive weeks (14 July through 28 July) corresponding to squaring, flowering and boll setting growth stage. The aim of reducing the data collection period was to determine a potential developmental window useful to practically evaluate the agronomic performance of numerous cotton candidate lines under irrigated and rainfed conditions in a breeding nursery.

Just before harvest, final plant height and the total number of main-stem nodes were measured from 10 plants in each plot. A random sample of 25 bolls was picked before harvest from each plot. Boll size was calculated by dividing seed cotton weight in grams by 25, and lint percent calculated by dividing lint weight by seed cotton weight. All tests were mechanically harvested using a two-row cotton stripper modified for small-plot harvesting with no burr extractor. Harvest weights were recorded for each plot, and a 600-g subsample of burr cotton was collected and ginned on a 10-saw laboratory gin with a stick machine, feeder-extractor and lint cleaner. Weighed lint percentage (gin turnout) from the subsample was used to calculate lint yield estimates from the plot harvest weights. A 10-g lint sample was collected from each ginned subsample and sent to the Texas Tech University Fiber and Biopolymer Research Institute for High Volume Instrument (HVI) fiber quality analysis.

### 4.4. Statistical Analysis

Data from irrigated and rainfed trials were analyzed separately using the general linear model (GLM) and mixed procedures of SAS, version 9.4. The GLM procedure was run with fixed effects to determine the relative magnitude of the main effect of genotype and year × genotype interactions. Because year × genotype interaction was not significant in GLM analysis, the SAS PROC MIXED procedure was applied to multiyear agronomic properties and fiber quality data by using replications and years as random effects.

For agronomic traits data analysis, weeks in different growth stages were included in the model both as the fixed effect and random effect to account for the agronomic data collected as a repeated measurement over time. Including week as a fixed effect in the model indicated how the average of the outcome changed over each week, while the random effects emphasized how much variability of the outcome that the plants had at each week. To validate normality and homoscedasticity of all measured variables, Shapiro–Wilk’s and Brown–Forsythe’s and Levene’s tests were used. When the data met the criteria for normality and homoscedasticity assumptions, all agronomic traits data, yield, and fiber quality traits were analyzed with SAS 9.4 (SAS Institute Inc). For non-normally-distributed count data, square root transformation was applied. Tukey’s HSD test was used to determine differences among genotypes for different traits at the *p* ≤ 0.05 level of significance. The least-square means were calculated using JMP Genomics 6 (JMP, 2013), where year and replication were treated as random effects. Correlation analysis was performed using the restricted maximum likelihood method to evaluate the relationship between different traits of interest.

## Figures and Tables

**Table 1:** Analysis of variance for the number of bolls (NB), number of squares (NSQR), number of flowers (NF), plant height (PH), and number of nodes (NN) for upland cotton lines grown under irrigated and rainfed conditions. DF—degrees of freedom.

Sources of Variance	DF	Irrigated					Rainfed				
		NSQR	NF	NN	NB	PH	NSQR	NF	NN	NB	PH
Genotypes	8	<0.0001 *	0.2164	<0.0001 *	<0.0001 *	<0.0001 *	<0.0001 *	0.3662	0.1144	<0.0001 *	<0.0001 *
Week	8	<0.0001 *	<0.0001 *	<0.0001 *	<0.0001 *	<0.0001 *	<0.0001 *	<0.0001 *	<0.0001 *	<0.0001 *	<0.0001 *
Genotypes * Week	64	0.0004 *	0.000 *	0.9938	0.0267 *	0.9909	0.6795	0.0232 *	0.6795	0.026 *	1.000

* Significant at the <0.05 probability level. Notes. Week represents the period across cotton plant growth stages. Data for agronomic traits were collected for nine consecutive weeks starting from 50 days after planting. Square root transformations were used for all counted traits.

**Table 2:** Least square means for the number of bolls per plant (NB), boll size, plant height (PH), lint turnout (LT %), and lint yield of cotton lines grown under irrigated and rainfed conditions.

Genotypes	Irrigated					Rainfed				
	Sqrt (NB)	PH (cm)	Boll Size (g)	LT (%)	Yield (kg ha^−1^)	Sqrt (NB)	PH (cm)	Boll Size (g)	LT (%)	Yield (kg ha^−1^)
06-46-153P	3.2 a ^†^	41 cd	5.5 a	22.9 e	972 cd	2.5 a	36 bcd	4.3 ab	23.6 cd	436 ab
11-21-703S	3.1 a	40 d	5.4 ab	27.2 cd	1156 b	1.9 abc	34 e	4.6 a	26.7 ab	465 a
12-8-103S	3.1 a	40 d	4.8 ab	23.1 e	1057 abc	1.8 abc	35 de	3.6 bc	22.2 d	387 bc
CS 50	2.7 ab	42 cd	3.9 c	29.9 a	1121 abc	2.2 ab	37 bcd	3.0 c	29.2 a	446 ab
DP 491	3.1 a	41 cd	4.9 ab	26.1 d	1101 bc	1.9 abc	36 b	4.1 ab	26.6 b	460 a
FM 989	3.0 a	43 c	5.3 ab	28.2 bc	1267 a	1.7 bc	37 bcd	3.8 b	26.3 b	434 ab
L23	2.7 ab	46 b	4.7 b	28.8 ab	1082 bc	1.5 c	38 bc	3.9 abc	27.6 b	410 ab
TX 1151	2.2 b	49 a	4.8 ab	21.4 f	652 e	1.5 c	42 a	3.5 bc	19.7 e	217 d
TX 62	2.1 c	41 cd	5.4 ab	23.7 e	826 d	1.4 c	38 b	4.6 a	24.1 c	341 c

Notes. Sqrt (NB): number of bolls-square root transformation was applied. PH: plant height cm; LT: lint turnout %; boll size: seed cotton weight in grams boll^−1^. ^†^ Means with the same letters are not significantly different at *p* < 0.05.

**Table 3:** Least square means for micronaire (no unit), upper half mean length (mm), length uniformity (%), strength (kN m kg -1), and elongation (%) of upland cotton lines grown under irrigated and rainfed conditions.

Genotypes	Irrigated					Rainfed				
	Micronaire	Length (mm)	Uniformity (%)	Strength (kN. m kg^−1^)	Elongation (%)	Micronaire	Length (mm)	Uniformity (%)	Strength (kN. m kg^−1^)	Elongation (%)
06-46-153P	3.8 ab ^†^	29.3 b	81.2 bcd	316.0 a	7.2 abcd	4.0 a	27.0 bc	79.8 ab	292.0 abc	6.8 abcd
11-21-703S	4.1 ab	29.3 b	81.7 ab	317.4 a	6.6 d	3.7 abc	27.1 bc	79.7 ab	291.4 abc	6.61 d
12-8-103S	3.8 ab	31.2 a	81.9 ab	328.0 a	7.3 abc	3.7 bc	29.2 a	79.6 b	319.1 a	7.3 ab
CS 50	3.9 ab	28.4 bc	80.7 d	303.2 b	7.0 bcd	4.1 a	26.1 cd	80.1 a	278.0 cd	6.7 bcd
DP 491	4.0 ab	27.8 c	81.3 bcd	309.6 ab	7.3 abc	3.9 ab	26.4 cd	78.8 bc	286.1 bcd	7.2 abc
FM 989	4.2 a	28.2 bc	81.2 bcd	315.4 ab	6.7 cd	3.9 ab	26.0 cd	79.5 ab	293.3 abc	6.9 abc
L23	3.9 ab	28.8 bc	80.9 cd	326.2 a	7.5 ab	4.0 ab	27.2 bc	79.8 ab	306.7 ab	7.3 abc
TX 1151	3.7 b	29.4 b	82.3 a	311.8 ab	6.6 cd	3.5 c	28.0 ab	78.8 bc	292.5 abc	6.6 c
TX 62	3.8 ab	27.6 c	79.5 c	276.5 c	7.7 a	3.6 bc	25.7 d	78.2 c	258.5 d	7.4 a

^†^ Means with the same letters are not significantly different at *p* < 0.05.

**Table 4:** Pearson’s correlation coefficient (r) showing the relationship between yield, agronomic traits and selected fiber properties of upland cotton lines grown under irrigated and rainfed conditions. Note: the upper half of the correlation table shows the relationship between different traits in rainfed trial, while the lower half of the correlation table shows the relationship between different traits in irrigated conditions.

**Irrigated**	**Rainfed**												
	Traits	Yield	BS	NSQR	NN	PH	NB	NF	Micronaire	Length	Uniformity	Strength	Elongation
	Yield	1	0.41 **	0.45 **	0.57 **	0.72 ***	0.74 ***	−0.16	0.50 **	0.30 *	0.51 **	0.53 **	0.40 **
	BS	−0.04	1	0.12	0.22	0.39*	0.34 *	−0.17	0.31 *	0.09	0.25	0.12	0.31 *
	NSQR	0.28 *	−0.28 *	1	0.56 **	0.40 **	0.44 **	0.27 *	0.50 **	0.17	0.33 *	0.40 **	0.01
	NN	0.33 *	−0.41 **	0.61 **	1	0.68 **	0.70 ***	−0.22	0.21	0.11	0.35 *	0.31 *	0.34 *
	PH	−0.03	−0.34 *	0.11	0.59 **	1	0.75 **	−0.30 *	0.37 *	0.31 *	0.48 **	0.42 **	0.47 **
	NB	0.16	−0.26 *	0.13	0.48 **	0.75 ***	1	−0.22	0.16	0.23	0.42 **	0.36 **	0.46 **
	NF	0.15	−0.35 *	0.38 *	0.43 **	0.18	0.33 *	1	0.25 *	−0.05	−0.15	−0.01	−0.36 *
	Micronaire	0.00	0.40 **	−0.33 *	−0.26 *	−0.27 *	−0.10	−0.34 *	1	0.16	0.33 *	0.35 *	−0.04
	Length	0.29 *	−0.04	0.29 *	0.20	−0.04	−0.05	0.17	−0.38 *	1	0.67 ***	0.79 ***	0.09
	Uniformity	0.13	0.34 *	−0.02	0.04	−0.05	0.01	−0.06	0.18	0.48 **	1	0.73	0.21
	Strength	0.38 *	−0.23	0.34 *	0.45 **	0.26 *	0.23	0.29 *	−0.38 *	0.66 **	0.34 *	1	0.12
	Elongation	−0.21	0.23	−0.41	−0.11	0.24	0.31 *	−0.17	0.29 *	−0.31	−0.07	−0.32	1

BS: boll size; NSQR: number of squares; NN: number of nodes; PH cm: plant height; NB: number of bolls; NF: number of flowers,* significance at *p <* 0.05; ** significant at *p*< 0.01; *** significant at *p* < 0.001.

**Table 5:** Total rainfall, growing degree days and the amount of irrigation water used in 2014, 2015 and 2016 growing seasons.

Months	2014		2015		2016	
	Total Rainfall	GDD_15.6_	Total Rainfall	GDD_15.6_	Total Rainfall	GDD_15.6_
	mm	°C	mm	°C	mm	°C
May	15	177	26	177	32	177
June	66	300	26	282	55	308
July	67	341	15	376	101	452
August	14	357	77	350	6	319
September	176	151	37	234	13	180
October	10	103	27	116	109	174
DD (May–October)		1429		1535		1610
Total Rainfall	348		208		316	
Total Irr	157		338		320	
Irr + Rainfall	505		546		636	

Notes. Irr: Irrigation; GDD15.6: growing degree days at 15.6 °C threshold for cotton. GDD15.6 were calculated based on the National Weather Service data of Lubbock, TX. using means of each maximum and minimum daily temperature for each month during the growing seasons of the cotton.
